# Short-Term and Sub-Chronic Dietary Exposure to Aspalathin-Enriched Green Rooibos (*Aspalathus linearis*) Extract Affects Rat Liver Function and Antioxidant Status

**DOI:** 10.3390/molecules201219868

**Published:** 2015-12-18

**Authors:** Johanna Debora van der Merwe, Dalene de Beer, Elizabeth Joubert, Wentzel C. A. Gelderblom

**Affiliations:** 1 Department of Food Science, Stellenbosch University, Private Bag X1, Matieland (Stellenbosch) 7602, South Africa; debora@factssa.com (J.D.M.); JoubertL@arc.agric.za (E.J.); 2 Post-Harvest and Wine Technology Division, Agricultural Research Council (ARC), Infruitec-Nietvoorbij, Private Bag X5026, Stellenbosch 7599, South Africa; DBeerD@arc.agric.za; 3 Institute of Biomedical and Microbial Biotechnology, Cape Peninsula University of Technology, P. O. Box 1906, Bellville 7535, South Africa; 4 Department of Biochemistry, Stellenbosch University, Private Bag X1, Matieland (Stellenbosch) 7602, South Africa

**Keywords:** herbal tea, biochemical parameters, serum iron, endogenous antioxidant enzymes, gene expression

## Abstract

An aspalathin-enriched green rooibos (*Aspalathus linearis*) extract (GRE) was fed to male Fischer rats in two independent studies for 28 and 90 days. The average dietary total polyphenol (TP) intake was 75.6 and 62.7 mg Gallic acid equivalents (GAE)/kg body weight (bw)/day over 28 and 90 days, respectively, equaling human equivalent doses (HEDs) of 12.3 and 10.2 GAE mg/kg bw/day. Aspalathin intake of 29.5 mg/kg bw/day represents a HED of 4.8 mg/kg bw/day (90 day study). Consumption of GRE increased feed intake significantly (*p* < 0.05) compared to the control after 90 days, but no effect on body and organ weight parameters was observed. GRE significantly (*p* < 0.05) reduced serum total cholesterol and iron levels, whilst significantly (*p* < 0.05) increasing alkaline phosphatase enzyme activity after 90 days. Endogenous antioxidant enzyme activity in the liver, *i.e.*, catalase and superoxide dismutase activity, was not adversely affected. Glutathione reductase activity significantly (*p* < 0.05) increased after 28 days, while glutathione (GSH) content was decreased after 90 days, suggesting an altered glutathione redox cycle. Quantitative Real Time polymerase chain reaction (PCR) analysis showed altered expression of certain antioxidant defense and oxidative stress related genes, indicative, among others, of an underlying oxidative stress related to changes in the GSH redox pathway and possible biliary dysfunction.

## 1. Introduction

Phytochemicals, such as plant polyphenols, are associated with beneficial effects in humans, but considerable scientific evidence exists to suggest that chronic consumption, specifically when consumed at high dose levels, may lead to adverse biological effects such as altering the cellular redox status due to their pro-oxidative activity [1]. Therefore, polyphenols is suggested to play a dual role, the outcome of which is determined by the levels and duration of exposure in conjunction with the physiological conditions of the cell [2].

Reactive oxygen species are produced from several reactions within a cell and are closely regulated by a multiple biological network of protection [3]. This network includes the antioxidant enzymes, catalase (CAT), superoxide dismutase (SOD) and glutathione peroxidase (GPx), and endogenous antioxidants such as glutathione (GSH), vitamins E and C, and the β-nicotinamide adenine dinucleotide phosphate (NADPH) associated glutathione reductase (GR), as well as exogenous antioxidants including plant polyphenols [4]. However, excessive exposure to polyphenols may overwhelm these antioxidant defense mechanisms in the cell, resulting in the over-production of reactive oxygen intermediates [2]. This ultimately contributes to cell injury and DNA damage, with the potential induction of irreversible pre-neoplastic lesions and carcinogenesis. Beneficial antioxidant properties can thus be outweighed by adverse effects and consequently the unregulated use of commercially available polyphenol-enriched foods or dietary supplements are of concern [2,5]. However, many uncertainties exist about the relative safe levels for such products.

The South African herbal tea, rooibos (*Aspalathus linearis*), has a very long history of traditional use and anecdotal evidence, known since the late 1960s, that rooibos alleviates infantile colic, has contributed greatly to general acceptance of its “healthy” image [6]. The increasing number of reports demonstrating beneficial biological effects for rooibos has shifted focus to its potential use as source material for production of nutraceutical extracts, in particular extract enriched with the dihydrochalcone, aspalathin, a flavonoid novel to rooibos [6,7]. Extracts containing high levels of aspalathin exhibit anti-diabetic properties [7,8,9,10,11,12], underpinning its potential use as a disease condition-specific nutraceutical. It therefore became essential to assess possible adverse biological effects associated with a high intake of polyphenol-enriched rooibos extracts.

The aim of the present study was to evaluate changes in the serum clinical biochemical parameters and oxidative parameters in rat liver following short-term (28 days) and sub-chronic (90 days) ingestion of an aspalathin-enriched green rooibos extract (GRE), shown to exhibit anti-diabetic effects in rats by Muller *et al.* [7]. For additional insights into the results obtained by monitoring the conventional serum clinical biochemical biomarkers for liver and kidney function, changes in expression of antioxidant defense and oxidative stress related genes, following the short-term consumption of GRE, were assessed in the liver for the first time.

## 2. Results and Discussion

### 2.1. Characterization of GRE

The total polyphenol content (TP) of GRE was 39.2 g Gallic acid equivalents/100 g extract. The total antioxidant capacity (TAC) of GRE, assessed using the ferric reducing antioxidant power (FRAP), (2,2-diphenyl-1-picrylhydrazyl (DPPH) radical scavenging, and oxygen radical absorbance capacity (ORAC) assays were 2128, 2774 and 12,989 μmol Trolox equivalents/g extract, respectively. HPLC data for the major rooibos flavonoids, present in GRE, were reported previously [7]. Briefly, the aspalathin content of the extract was 18.4% and the other flavonoids quantified, comprised 7.3% of the extract. These include the flavone derivatives of aspalathin, isoorientin and orientin, the dihydrochalcone, nothofagin and its flavone derivatives, isovitexin and vitexin, and the quercetin glycosides, quercetin-3-*O*-robinobioside, rutin, hyperoside and isoquercitrin. The extract also contained a phenylpropenoic acid glucoside, *i.e.* enolic phenylpyruvic acid 2-*O*-β-d-glucoside (PPAG), at 0.5%.

### 2.2. Feed Intake Parameters, Body Weight Gain and Relative Organ Weights

No deaths or obvious clinical signs were observed when conducting the short-term (28 day) and sub-chronic (90 day) studies. The average feed, GRE and TP intake over the 28 day period was significantly (*p* < 0.05) higher when compared to the 90 day study (Table 1). The body weight gain and relative liver weights were significantly (*p* < 0.05) lower after 90 days compared to the 28 day feeding period, while the relative kidney weights were significantly (*p* < 0.05) higher. GRE significantly (*p* < 0.05) increased the average feed intake after 90 days compared to the control, while no effect on the feed intake and different body and organ weight parameters were noticed

**Table 1:** Average feed, GRE and TP intake, as well as relative liver and kidney weights after dietary consumption of GRE ^a^ by male Fischer rats for 28 and 90 days.

Duration	Feed Intake (g/100 g bw)	GRE ^a^ Intake (g/100 g bw)	TP Intake ^b^	Bwg (g)	RLW (%) ^c^	RKW (%) ^c^
**28 days**						
Control	9.73 ± 0.42a	-	- ^d^	94.50 ± 10.20a	3.57 ± 0.18a	0.65 ± 0.03a
GRE	9.65 ± 0.39a	0.019 ± 0.001a	7.57 ± 0.30a	89.50 ± 12.94a	3.70 ± 0.18a	0.63 ± 0.03a
**90 days**						
Control	7.27 ± 0.49b	-	- ^d^	126.85 ± 19.21b	2.73 ± 0.27b	0.80 ± 0.14b
GRE	7.97 ± 0.57c	0.016 ± 0.001b	6.27 ± 0.30b	139.86 ± 17.41b	2.68 ± 0.20b	0.75 ± 0.07b

Values represent the mean ± SD of ten rats per group and means in a column followed by different letters differ significantly (*p* < 0.05); ^a^ Feed mixture contained 2.0 g GRE extract/kg; ^b^ mg Gallic acid equivalents/100 g bw/day; ^c^ % bw, ^d^ TP content of control diet not assessed. *Abbreviations:* bw, body weight; bwg, bodyweight gain; GRE, green rooibos extract; RKW, relative kidney weight; RLW, relative liver weight; TP, total polyphenol.

The comparative intake of the individual rooibos polyphenols and PPAG is summarized in Table 2, with aspalathin, the major rooibos flavonoid, consumed at 3.56 and 2.95 mg/100 g bw/day in the 28 and 90 day studies, respectively.

**Table 2:** Average daily flavonoid and phenylpropenoic acid intake during the 28 and 90 day GRE ^a^ feeding studies in male Fisher rats (*n* = 10).

Structure	Compound	Substitution	Daily Intake ^b^ (28 days)	Daily Intake ^b^ (90 days)
Dihydrochalcones 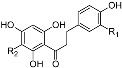	aspalathin	R_1_ = OH, R_2_ = β-d-glucopyranosyl	3.557 ± 0.14	2.947 ± 0.2
	nothofagin	R_1_ = H, R_2_ = β-d-glucosylpyranosyl	0.249 ± 0.01	0.207 ± 0.014
Flavones 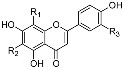	orientin	R_1_ = β-d-glucopyranosyl, R_2_ = H; R_3_ = OH	0.203 ± 0.01	0.17 ± 0.011
	isoorientin	R_1_ = H; R_2_ = β-d-glucopyranosyl, R_3_ = OH	0.396 ± 0.016	0.328 ± 0.022
	vitexin	R_1_ = β-d-glucopyranosyl, R_2_, R_3_ = H	0.052 ± 0.002	0.043 ± 0.003
	isovitexin	R_1_, R_3_ = H, R_2_ = β-d-glucopyranosyl	0.075 ± 0.003	0.06 ± 0.004
Flavonols 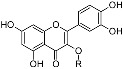	rutin	R = α-l-rhamnopyranosyl-(1→6)-β-d-glucopyranosyl (rutinosyl)	0.103 ± 0.004	0.086 ± 0.006
	quercetin-3-*O*-robinobioside	R = α-l-rhamnopyranosyl-(1→6)-β-d-galactopyranosyl (robinobiosyl)	0.20 ± 0.008	0.17 ± 0.011
	hyperoside	R = β-d-galactopyranosyl	0.051 ± 0.002	0.043 ± 0.003
	isoquercitrin	R = β-d-glucopyranosyl	0.073 ± 0.003	0.060 ± 0.004
Phenylpropenoic acid 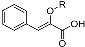	Enolic phenylpyruvic acid-2-*O*-β-d-glucopyranosyl	R = β-d-glucopyranosyl	0.095 ± 0.004	0.078 ± 0.005

^a^ Feed mixture contained 2.0 g GRE extract/kg; ^b^ Values calculated from GRE polyphenol content [7] and feed intake (Table 1) and expressed as mg compound/100 g body weight. *Abbreviations:* GRE, green rooibos extract; TP, total polyphenol.

### 2.3. Serum Clinical Biochemical Parameters

**Figure 1:**
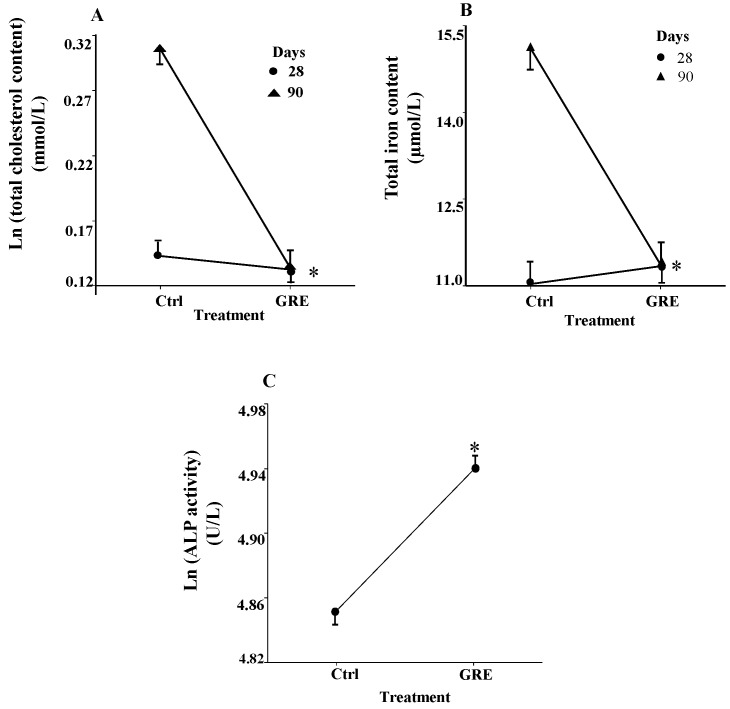
Modulation of serum clinical biochemical parameters of male Fischer rats by GRE following a dietary treatment of 28 (*n* = 10 per group) and 90 days (*n* = 10 per group), respectively. (**A**) Interactive effects of GRE on Chol levels after 28 and 90 days; (**B**) interactive effect of GRE on Fe levels after 28 and 90 days; and (**C**) GRE main effect on ALP. Plots of Chol and ALP derived from log transformed data (means ± standard error). * GRE differs significantly when compared to the 90 day control treatment (*p* < 0.05 for (**A**); *p* < 0.001 for (**C**)); GRE differs significantly (*p* < 0.05) from the control (main effect—(**C**)). *Abbreviations*: Chol, total cholesterol; ALP, alkaline phosphatase; serum Fe, total iron.

The extract did not significantly affect the liver function enzymes after the 28 day feeding period (Table 3). Rats fed for 90 days, irrespective of treatment, had significantly (*p* < 0.05) higher serum levels of alanine transaminase (ALT), unconjugated bilirubin (Dbili) (only compared to GRE treatment for 28 days) and creatinine compared to the rats fed for 28 days. On the other hand, their total bilirubin (Tbili) levels were significantly lower after 90 days compared to 28 days (Table 3). Significant interactions were observed for serum cholesterol (Figure 1A; *p* < 0.05) and iron (Figure 1B; *p* < 0.001) indicating that GRE reduced their levels after 90 days to a level similar to the 28 day treatment. The GRE significantly (*p* = 0.03) increased ALP in the serum when compared to the control treated rats (Figure 1C).

**Table 3:** Serum clinical biochemical indicators of liver function after dietary consumption of GRE ^a^ by male Fischer rats for 28 and 90 days.

Clinical Parameter	GGT (U/L)	ALT (U/L)	AST (μmol/L)	Tbili (μmol/L)	Dbili (μmol/L)	Creat (μmol/L)
28 days						
Control	2.70 (2.75)a	44.20 (9.90)a	104.90 (21.93)a	8.26 (1.75)a	0.99 (0.41)ab	46.19 (4.54)a
GRE	1.80 (2.70)a	42.40 (5.04)a	105.40 (12.96)a	7.78 (1.21)a	0.84 (0.21)a	46.09 (2.72)a
90 days						
Control	3.00 (1.89)a	76.33 (7.57)b	107.80 (25.73)a	2.46 (0.36)b	1.24 (0.23)b	64.70 (8.41)b
GRE	3.67 (2.00)a	83.00 (14.02)b	121.00 (16.17)a	2.09 (0.22)b	1.30 (0.33)b	60.33 (3.77)b

Values represent the mean ± SD (values in brackets) of ten rats per group; Means in a column followed by different letters differ significantly (*p* < 0.05); ^a^ Feed mixture contained 2.0 g GRE extract/kg. *Abbreviations:* GRE, green rooibos extract; GGT, gamma-glutamyl transferase activity; ALT, alanine transaminase; AST, aspartate transaminase; Tbili total bilirubin; Dbili unconjugated bilirubin; Creat, creatinine.

### 2.4. Oxidative Parameters in the Liver

**Figure 2:**
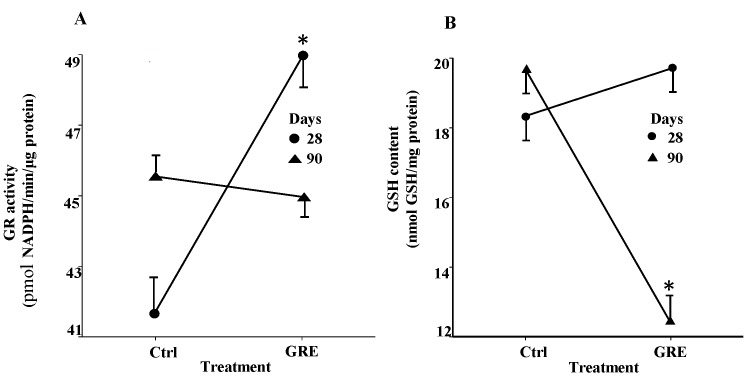
Interactive effects of GRE on GR activity (**A**) and GSH levels (**B**) in the liver of male Fischer rats fed over a period of 28 (*n* = 10 per group) and 90 (*n* = 10 per group) days, respectively. * Values are means ± standard error and differ significantly (*p* < 0.05) when compared to the control treatment. *Abbreviations:* GR, glutathione reductase; GRE, green rooibos extract; GSH, glutathione; NADPH, β-nicotinamide adenine dinucleotide phosphate.

GRE consumption did not significantly (*p* ≥ 0.05) alter the activity of the antioxidant liver enzymes, CAT and SOD, compared to the respective controls at 28 and 90 days (Table 4). GR activity was, however, significantly (*p* < 0.05) elevated following the 28 day GRE treatment, but not for the 90 day treatment (Figure 2A). In contrast, the GSH concentration in the liver was not significantly (*p* ≥ 0.05) affected by GRE after 28 days, but after 90 days its activity was significantly (*p* < 0.05) reduced compared to the control (Figure 2B). No significant (*p* ≥ 0.05) effect was noted in the oxidized glutathione (GSSG) levels and GSH/GSSG ratio, irrespective of treatment period (Table 4). Similarly, GRE had no significant (*p* ≥ 0.05) effect on the lipid peroxidation parameters, conjugated diene and malondialdehyde levels, in the liver.

**Table 4:** Effect of dietary consumption of GRE ^a^ on oxidative stress liver parameters of male Fischer rats after 28 and 90 days.

Parameter	28 days		90 days	
	Control	GRE	Control	GRE
CAT ^b^	320.69 ± 36.85a	296.56 ± 28.56a	323.47 ± 33.29a	320.85 ± 37.48a
SOD ^c^	0.64 ± 0.06a	0.58 ± 0.06a	0.55 ± 0.09a	0.53 ± 0.06a
GSSG ^d^	1.40 ± 0.42a	1.66 ± 0.57a	1.68 ± 0.33a	1.26 ± 0.37a
GSH/GSSG ^e^	12.80 ± 4.44a	11.95 ± 5.77a	11.35 ± 3.79a	9.66 ± 3.01a
CD ^f^	10.43 ± 2.08a	10.20 ± 2.13a	10.57 ± 1.68a	12.27 ± 1.44a
MDA ^g^	1.58 ± 0.53a	1.67 ± 0.66a	1.50 ± 0.59a	1.78 ± 0.43a

Values represent the mean ± SD of duplicate analysis of five random samples selected from each treatment group (*n* = 10) and means in the same row per study followed by different letters are significantly different (*p* < 0.05). ^a^ Feed mixture contained 2.0 g GRE extract/kg; ^b^ activity in nmole H_2_O_2_/min/μg protein; ^c^ activity given as the amount of protein (μg) required to produce a 50% inhibition of auto-oxidation of 6-hydoxydopamine; ^d^ nmole/mg protein; ^e^ GSH/GSSG ratio; ^f^ nmole/mg protein; ^g^ μmol/mg protein. *Abbreviations:* CAT, catalase; CD, conjugated dienes; GRE, green rooibos extract; GSH, glutathione; GSSG, oxidized glutathione; MDA, malondialdehyde; SOD, superoxide dismutase.

### 2.5. Differential Expression of Antioxidant Defense and Oxidative Stress Related Genes

Quantitative Real Time-PCR array analysis of the relative expression of 84 oxidative stress and antioxidant defense genes in rat liver was conducted after 28 days of treatment. No analyses were conducted after 90 days as the 28 days exposure was consider sufficient to effect changes regarding these genes in the liver. Significant (*p* < 0.05) changes detected in the expression of seven genes as a result of GRE intake are summarized in Table 5.

**Table 5:** Fold change ^a^ and *p*-values of the genes affected by dietary consumption of GRE ^b^ during the 28 study with male Fischer rats (*n* = 3 per group).

Function Grouping and Name of Gene	Symbol	FC ^c^	*p*-Value ^d^
(i) Antioxidant defense related genes			
*Glutathione Peroxidases*			
Glutathione peroxidase 2	*Gpx2*	+1.80 ^c^	0.04
Glutathione peroxidase 3	*Gpx3*	−1.20 ^d^	0.04
(i) Genes involved in reactive oxygen species (ROS) metabolism			
*Oxidative Stress Responsive Genes*			
Aminoadipate-semialdehyde synthase	*Aass*	+1.32	0.01
Apolipoprotein E	*Apoe*	−1.27	0.05
Isocitrate dehydrogenase 1	*Idh1*	+1.30	0.04
NAD(P)H dehydrogenase, quinone 1	*Nqo1*	+1.68	0.02
*Other genes involved in superoxide metabolism*			
Neutrophil cytosolic factor 2	*Ncf2*	−4.78	0.03

^a^ Values preceded by (+) and (−) indicate up- and down-regulation, respectively; ^b^ Feed mixture contained 2.0 g GRE extract/kg; ^c^ FC indicates the fold change, ^d^ *p* < 0.05 designated as significant.

GRE significantly (*p* < 0.05) affected the expression of two genes encoding for antioxidant defense, including the 1.8 fold up-regulation of glutathione peroxidase 2 (*Gpx2*) and a 1.2 fold down-regulation of *Gpx3*.

GRE significantly (*p* < 0.05) up-regulated some of the oxidative stress responsive genes including aminoadipate-semialdehyde synthase (*Aass*), isocitrate dehydrogenase 1 (*Idh1*) and NAD(P)H dehydrogenase, quinone 1 (*Nqo*1) 1.32, 1.30 and 1.68 fold, respectively, while the expression of apolipoprotein E (*Apoe*) was down-regulated 1.27 fold (*p* = 0.05). Neutrophil cytosolic factor 2 (*Ncf2*), involved in superoxide metabolism, was down-regulated 4.78 fold by GRE.

GRE treatment did not significantly (*p* ≥ 0.05) affect any of the genes for oxygen transporters.

Studies in cell cultures and experimental animals indicated that antioxidants may cause adverse toxic effects, particularly when administered or consumed at high dose levels. Studies on flavanol-enriched green tea (unfermented *Camellia sinensis* (*C. sinesis*)) preparations, containing mainly the potent antioxidant, (−)-epigallocatechin gallate (EGCG), have been reported to exhibit hepato- and nephrotoxicity effects when administered as a single high dose to mice (1500 mg/kg), rats (2000 mg/kg) and dogs (1500 mg/kg) [13,14]. In humans, consumption of high doses of tea-based (*C. sinensis*) dietary supplements showed elevated ALT and Tbili levels, which were resolved following cessation of the supplement consumption [15]. It became necessary that investigations on specific health effects of antioxidants should incorporate studies to establish “safety levels” to avoid toxic or adverse health effects [16].

No effect on the body weight gain and the relative liver and kidney weights of the rats was observed as a result of the 28 and 90 day GRE treatments. Although the baseline Tbili was higher in the younger rats and Dbili, ALT and creatinine levels increased significantly (*p* < 0.05) in the older rats, these parameters were not altered by GRE. A significant (*p* = 0.03) increase in ALP was evident in the rats chronically exposed to GRE. Although increased serum ALP is associated with drug-induced cholestasis [17], the gamma glutamyl-transferase (GGT) activity, considered to be a more reliable marker for cholestasis [18], was not significantly (*p* ≥ 0.05) increased by GRE. A specific role of the rooibos flavonoids in the disruption of biliary function is not known at present and different manifestations thereof could prevail depending on the age of the rats and the level and duration of GRE exposure.

An underlying oxidative stress, however, seems to prevail after 28 days when considering the expression of the oxidative stress and antioxidant response genes. This is despite the fact that none of the oxidative stress markers of lipid peroxidation in the liver, *i.e.*, conjugated dienes and malondialdehyde, were significantly (*p* ≥ 0.05) altered. This could imply a lack of sensitivity of these markers when considering oxidative stress. In this regard the respective up- and down-regulation of *Gpx2* and *Gpx3* involved in hydrogen peroxide degradation is of particular interest. The *Gpx2* gene is also a target for NF-E2-related factor 2 (Nrf2), a transcription factor that regulates important antioxidant and phase II detoxifying genes [19]. *Gpx2* suppresses cyclooxygenase activity by removal of hydroperoxides required for enzyme activation, thereby facilitating an anti-inflammatory function [20]. The up-regulation of *Gpx2* by GRE may indicate an anti-inflammatory response as a result of an underlying oxidative stress due to the high levels of flavonoids consumed. Down-regulation of *Gpx3* by GRE could facilitate oxidative stress due to reduced ROS quenching. This gene is down-regulated during neoplastic transformation as compared to healthy tissue, where it presumably plays a role as a tumor suppressor [20] and therefore its down-regulation may be unfavorable during various stages of cancer development. Of interest is the up-regulation of peroxidase, *Aass*, encoding a catalyzing peroxidase protein enzyme that plays an antioxidant protective role in cells and is involved in lysine degradation [21]. The latter is known to cause lipid peroxidation, reducing the level of GSH and gluthathione peroxidases, thereby impairing antioxidant defenses mechanisms. It would appear that the hyper-expression of the *Aass* gene is associated with protection of the liver against the harmful action of ROS involving the GSH redox cycle. Although the activity of the antioxidant enzymes, CAT and SOD, were not altered, GR activity was significantly (*p* < 0.05) increased after 28 days by GRE, while it reduced the GSH level in the liver after 90 days, suggesting modulation of the GSH redox cycle. The decrease in GSH is therefore of relevance as polyphenols are known to be an important determining factor when considering their pro-oxidant activity and their interaction with glutathione metabolism [22,23,24,25]. It is known that monophenol-type flavonoids is prone to cause GSH oxidation, while flavonoids with a catechol group on the B-ring may lead to GSH conjugation. GSH oxidation seems not to be involved, since the GSSG level was not altered. The formation of GSH conjugates via flavonoid quinone formation is therefore more likely. Studies in hepatocytes indicated that flavonoids with the catechol group possessing low redox potentials, such as luteolin and quercetin, depleted hepatocyte GSH (Galati *et al.*, 2002). Therefore, despite the increase in expression of *Gpx2*, an increase in the GSSG levels was not observed, which could be related to the reduction in the GSH levels via the interaction with the rooibos flavonoids. Of interest was that the GR activity was significantly increased after 28 days, but tended to stabilize after 90 days. GSH depletion has been proposed as a potential strategy to sensitize the cell to phenoxyl radical-induced oxidative stress and mitochondrial membrane potential collapse [26]. However, it may adversely affect the GSH redox cycle under normal physiological conditions due to the potential pro-oxidant activity [24,25,27]. In this regard, pro-oxidant activity of rooibos aqueous extracts, their crude polymeric fractions and pure aspalathin has been reported while “fermentation” (*i.e.*, oxidation) of rooibos decreased the pro-oxidant activity associated with the concomitant decrease in the aspalathin content [28]. Major GRE flavonoids containing a B-ring catechol arrangement are aspalathin, isoorientin, orientin and the quercetin glycosides, *i.e.*, rutin, isoquercitrin and quercetin-3-*O*-robinobioside.

Further evidence towards the possible induction of oxidative stress by GRE is the up- and down-regulation of genes involved in ROS metabolism. These include the down-regulation of *Ncf2/p67phox* and *Apoe* genes and the up-regulation of *Ncf2*, *Idh1* and *Nqo1* genes. The role of *Ncf2* in the liver is unclear, however, it encodes for a multi-enzyme complex known as NADPH oxidase, which plays an essential role in regulating the activity of neutrophils [29,30]. NADPH oxidase produces superoxide anion and other ROS from molecular oxygen, using NADPH as electron donor and influences a multitude of biological functions including host defense and redox signaling [30,31]. NADP^+^-dependent isocitrate dehydrogenase (Idh1) also provides NADPH needed for the regeneration of GSH, as well as for fat and cholesterol synthesis [32]. In addition, down-regulation of apolipoprotein E (*Apoe*) gene expression may also impact on cholesterol homeostasis as Apoe plays a key role in metabolism of cholesterol and triglycerides by binding to receptors in the liver, contributing to the clearance of chylomicrons, very low density lipoprotein (VLDL) and high density lipoprotein (HDL) from plasma [33]. In contrast, the up-regulation of *Idh1* and the corresponding increase in the associated protein, Idh1, are therefore of relevance [34]. In normal cellular metabolism, Idh1 plays an important role in lipid metabolism, maintaining cellular cholesterol and fatty acid homeostasis through synthesis and degradation. The enzyme is therefore suggested to be a target enzyme for lipid-lowering pharmacological strategies [35]. An association with cellular response to oxidative insults and an increased activity of the enzyme have also being suggested [34]. Dysregulation of *Idh1* is a common phenomenon in cancer cells as it functions at a crossroad of cellular metabolism in lipid synthesis and cellular defense against oxidative stress and cancer cells may gain from the glucose sensing role of Idh1 [34]. The outcome of the up-regulation of *Idh1* expression by GRE will therefore depend on the specific conditions in terms of oxidative stress and disease. Changes in the expression of these genes could therefore be related to the lowering of serum cholesterol, as well as the reduction in the GSH level in the liver. Subsequent studies should focus on the effect of GRE on the translation protein levels of the associated enzymes. This is of specific interest, as a study in humans who consumed six cups of fermented rooibos daily for six weeks had decreased LDL-cholesterol and increased HDL-cholesterol and GSH levels in the blood [36].

The *Nqo1* gene is a member of the NAD(P)H: quinone oxidoreductase family that prevents the one electron reduction of quinones resulting in the production of radical species. The up-regulation of the gene by GRE suggested it to be part of an oxidative stress response and it is reported to be overexpressed in certain types of malignant tissues in the colon, breast, lung and liver [37]. Therefore the increased expression of this gene in the liver following GRE consumption is of concern and warrants further investigation. The expression of the *Nqo1* gene, as was mentioned for the *Gpx2* gene, is highly regulated by *Nrf2* directly via an antioxidant response element (ARE) and plays an integral role in cellular responses to oxidative stress [38]. In mice electrophilic chemicals up-regulated the expression of *Nqo1* in an Nrf2-dependent fashion [39]. The rooibos flavone, isoorientin, up-regulates the expression of *Nqo1* via *Nrf2* in HepG2 cells, which was associated with an increased level of the antioxidant enzyme proteins [40]. The so-called “protective” effect resulting from up-regulated expression of *Nqo1* and *Gpx2* under the current study conditions could be related to a protective response towards GRE-induced oxidative stress in the liver.

The modulation of the oxidative status in the liver by rooibos flavonoids therefore seems to depend on the dose and the duration of GRE exposure. The current GRE preparation contained 18.4 g aspalathin/100 g extract, approximately 1.3–3 fold the concentration of aqueous unfermented (“green”) rooibos extracts, while the levels of nothofagin and the flavone glucosides, isoorientin, vitexin and isovitexin, were also increased [7,8]. The TP content of GRE (39.22 g GAE/100 g extract) was higher than the averages of 35.08 and 35.12 g GAE/100 g extract reported for aqueous extracts of unfermented rooibos by Joubert *et al.* [41] and de Beer *et al.* [42], respectively. Increased polyphenol content of GRE resulted in increased antioxidant activity in the FRAP, DPPH and ORAC assays when compared to aqueous unfermented rooibos extracts [42]. The increased activity is mainly attributed to the high aspalathin content, known to be a potent rooibos antioxidant when compared to the radical scavenging activity of quercetin and EGCG [43].

A 10 week study with fermented and unfermented rooibos as sole source of drinking fluid significantly (*p* < 0.05) reduced GSSG levels in the liver of rats, while GSH was markedly increased, resulting in a significant (*p* < 0.05) increase in the GSH/GSSG ratio [44]. Differences in the TP intake need to be considered as the dietary intake of 6.2 mg GAE/100 g bw/day for the current study was 2.5 times lower than that of the 10 week study when unfermented rooibos was consumed (16.2 mg GAE/100 g bw/day). The low TP exposure for 10 weeks increased the GSH level while in the current study, the lower dose over a period of 90 days reduced the GSH level. A three-week study in rats indicated that fermented rooibos as sole drinking fluid prevented lipid oxidation and oxidative stress effected by the hepatotoxic carcinogen, fumonisin B_1_ in rats at a TP exposure level of 6.4 mg GAE/100 g bw/day [44,45]. However, unfermented rooibos (TP of 16.1 mg GAE/100 g bw/day) synergistically increased the hepatotoxic effect of fumonisin B_1_ [45], which was attributed to pro-oxidant effects as demonstrated for rooibos extracts and aspalathin [28]. Both the fermented and unfermented rooibos significantly (*p* < 0.05) reduced the FB_1_-induced increase in the liver GSH level. In the current study an underlying oxidative stress appears to exist in rat liver following the consumption of GRE mainly via disruption of the GSH redox cycle.

Of interest was the significant (*p* < 0.001) decrease in the total serum iron levels by GRE after the 90 day dietary treatment, while no effect was evident after 28 days. No adverse effects on the serum Fe level was recorded at a three-fold lower TP level in the 10 week study using aqueous extract of unfermented rooibos as sole drinking fluid [44]. Disruption of iron absorption from the gut at a chronic high dose of exposure could have important implications in humans regarding physiological conditions associated with anemia. The high levels of TP consumed and the formation of non-transportable polyphenol-iron complexes [46] appears to be responsible for reducing the serum iron levels in the current study. However, studies in humans fail to provide evidence of a reduction in serum iron following consumption of fermented rooibos infusions thus far [36,47,48]. Daily consumption of six cups of fermented rooibos for six weeks did not affect the total serum iron levels of the subjects [36]. Based on the TP content of one cup (200 mL) of rooibos infusion and an average bw of 70 kg, the study subjects were exposed to an average of 5 mg TP/kg bw/day [36]. This is approximately 12.5 fold lower when compared to the current rat study (62.7 mg/kg bw/day). The difference in the exposure to aspalathin, the major rooibos antioxidant, was even greater (295 fold), considering that the rats consumed 29.5 mg aspalathin/kg bw/day compared to 0.1 mg aspalathin/kg bw/day for humans. The aspalathin content of the rooibos infusions in the human study was not quantified, but analysis of a large number of rooibos production batches, collected over three seasons, provided the expected average aspalathin content of a cup of rooibos infusion (1.2 mg aspalathin/200 mL) [49]. The dose of aspalathin received by the rats in the current study (90 days) translates to an human equivalent dose of approximately 4.8 mg/kg bw/day based on the body surface area normalization method [50]. Therefore, a threshold of exposure to rooibos polyphenols seems to exist when considering possible adverse and/or positive health outcomes. The current study provided evidence that consumption of high levels of an aspalathin-enriched rooibos extract could lead to some adverse effects in the liver, but more research is needed to determine the total polyphenol and/or aspalathin thresholds for these outcomes. Subsequent studies should focus on the protein levels of the specific genes that were upregulated to provide better insight into subtle changes related to the redox status in the liver and the kidneys effected by the polyphenol-enriched rooibos flavonoids.

## 3. Experimental Section

### 3.1. Green Rooibos Extract

Polyphenol-enriched green rooibos extract (GRE), previously characterized for phenolic content by HPLC-DAD and used by Muller *et al.* [7], was selected for the current study.

### 3.2. Chemicals

Analytical grade solvents and chemicals, purchased from Merck Millipore (Darmstadt, Germany) or Sigma-Aldrich (St. Louis, MO, USA), were used except if stated otherwise. TRIS (N_2_C(CH_2_OH)_3_ was obtained from Amersham Biosciences (Cleveland, OH, USA), while Triton-X-100 was supplied by BDH Chemicals Ltd. (Poole, UK). BCA protein assay reagent A (containing sodium carbonate, sodium bicarbonate, BCA detection reagent, and sodium tartrate in 0.1 N sodium hydroxide) and BCA protein assay reagent B were purchased from Separations Scientific (Cape Town, South Africa).

### 3.3. Total Polyphenol (TP) Content and Total Antioxidant Capacity (TAC) of GRE

The TP content and TAC of GRE were determined in triplicate in 96-well microplates, using a BioTek SynergyHT microplate reader (Winooski, VT, USA). The TP content was determined according to the Folin–Ciocalteu method adapted to microplate scale [51] and expressed as g Gallic acid equivalents (GAE)/100 g extract. The TAC of the extract was determined using the 2,2-diphenyl-1-picrylhydrazyl (DPPH) radical scavenging, ferric reducing antioxidant power (FRAP) and oxygen radical antioxidant capacity (ORAC) assays. The DPPH and FRAP assays were performed as described by Arthur *et al.* [51], while the ORAC assay was performed as described by Huang *et al.* [52]. For the latter assay, a thermal barrier was created by filling a single row of the outside wells with 300 μL water. All TAC values were expressed as μmol Trolox equivalents (TE)/g extract.

### 3.4. Short-Term (28 Day) and Sub-Chronic (90 Day) Feeding Studies in Male Fischer 344 Rats

#### 3.4.1. Animals and Diets

Male Fischer 344 rats (weighing between 150 and 200 g; 7–8 weeks old) were obtained from the Primate Unit of the Medical Research Council of South Africa. Animals were individually housed in stainless steel wire-bottomed cages fitted with Perspex houses in a closed environment (24–25 °C; 50% humidity) with a 12 h light-dark cycle. Feed cubes (Epol Ltd., Cape Town, South Africa) were milled, GRE mixed into the resulting mash (2.0 g GRE/kg mixture) and stored under nitrogen at −20 °C. Dose selection was based on a similar toxicity study conducted with green tea extracts in rats with a gavage dosage regimen of 1 mg/kg over a period of 14 weeks [53]. Due to differences in toxicokinetic profiles of chemicals between gavage and dietary exposure [54], a higher dose (up to 2 mg/kg) of the GRE was selected in the current study. The use of experimental animals was approved by the Ethics Committee for Research on Animals (ECRA) of the Medical Research Council of South Africa (ref. 05/07).

#### 3.4.2. Experimental Design and Sample Collection

The GRE-mash mixture was fed ad libitum to the rats for 28 and 90 days in two separate experiments. Rats were randomly divided into the two treatment and two control groups (10 rats/group) for the 28 day and 90 day studies. Feed intake was monitored every second day and the bw recorded weekly. The rats of the 28 day study were fasted overnight and terminated by cervical dislocation, blood collected and livers harvested, weighed and quickly frozen in liquid nitrogen. Blood was collected in BD Vacutainer SST II advance (BD, Plymouth, UK) tubes and serum prepared by centrifugation at 2000× *g* for 10 min at 4 °C and stored at −80 °C until analyzed. Upon sacrifice, a sample from each liver was rapidly snap-frozen in liquid nitrogen and stored at −80 °C for investigating changes in the gene expression profiles. Animals in the 90 day study were fasted overnight prior to termination and sacrificed under pentobarbital anesthesia. Blood were collected from the abdominal aorta and the liver harvested, weighed, frozen in liquid nitrogen and stored at −80 °C. Five liver samples were randomly selected from each treatment and control group of both studies and liver homogenates were prepared for biochemical analysis.

#### 3.4.3. Serum Clinical Biochemical Parameters

Clinical biochemical parameters, including total cholesterol and Fe, ALP, ALT, GGT, AST, Tbili, Dbili, creatinine, and total protein levels were determined using a Technicon RA 1000 automated analyzer at the Nutritional Intervention Research Unit of the Medical Research Council of South Africa (MRC, Bellville, South Africa).

#### 3.4.4. Antioxidant Enzymes Assays

Liver homogenates were prepared on ice by homogenizing ±200 mg of tissue in 2 mL of sodium phosphate buffer (50 mM, 0.5% (*w*/*v*) Triton X-100, pH 7.5) using five strokes of a Teflon Potter–Elvehjem homogenizer. The homogenates were sonicated for two 15 s bursts, followed by centrifugation at 15,000× *g* for 10 min at 4 °C. Homogenates were stored at −80 °C until analyzed. The protein concentration of the liver homogenates was determined in duplicate according to the method of Kaushal and Barnes [55], using bovine serum albumin (BSA) as standard.

CAT activity was determined by monitoring the decomposition of hydrogen peroxide at 240 nm according to the method described by Aebi [56]. Results were expressed as nmol H_2_O_2_/min/μg protein and represent the mean of duplicate determinations. SOD activity was determined as described by Marnewick *et al.* [45]. The liver homogenate samples were diluted to a concentration of 0.1 μg protein/μL in sodium phosphate buffer (50 mM, pH 7.4). The SOD activity was expressed as the amount of protein (ng) required to produce a 50% inhibition of auto-oxidation of 6-hydroxydopamine and represent the mean of duplicate determinations. GR activity, measured as the oxidation of nicotinamide adenine dinucleotide phosphate (NADPH), was determined in duplicate and activity was expressed as pmole NADPH used/min/μg protein according to Ellerby and Bredesen [57].

#### 3.4.5. Glutathione Analysis

Liver tissue (250 mg) was ground in liquid nitrogen [58] and protein determined (20 mg) with BSA as standard according to the method described by Markwell *et al.* [59]. For GSH determination the ground sample (100 mg) was deproteinized using 1 mL 15% (*w*/*v*) trichloroacetic acid (TCA) containing ethylenediaminetetra-acetic acid (EDTA; 1 mM). For GSSG determination ground sample (100 mg) was homogenized in 1 mL 6% (*v*/*v*) HClO_4_, containing freshly prepared 3 mM M2VP (dissolved in 0.1 N HCl and EDTA (1 mM) on ice, using a Teflon Potter–Elvehjem homogenizer. Samples were stored at −80 °C until analyzed.

GSH and GSSG were determined as described by Abel *et al.* [60] with some modifications. The homogenates were centrifuged at 15,000× *g* for 10 min at 4 °C and the supernatant diluted with 0.5 M sodium phosphate buffer containing 1 mM EDTA (pH 7.5) at 1:600 for GSH and 1:30 for GSSG. For the assay, 50 μL of diluted samples or standards, 50 μL 5,5-dithiobis-(2-nitrobenzoic acid) (DTNB) and 50 μL GR from *Saccharomyces cerevisiae* (baker’s yeast) stock solution were added to the respective wells of Greiner 96-well clear flat-bottom polystyrene plates. The final reaction mixture contained 0.25 U GR, 0.15 mM DTNB and 0.25 mM NADPH in sodium phosphate buffer. The concentration of GSH and GSSG in samples was determined by using a standard curve (0.5–3 μM) for each and the results expressed as nmole GSH or GSSG/mg protein.

#### 3.4.6. Lipid Peroxidation in Rat Liver Homogenates

Liver samples were homogenized on ice in 19 volumes potassium phosphate buffer (10 mM, 1.15% KCl, pH 7.4) using a Teflon Potter–Elvehjem homogenizer. The homogenates were stored at −80 °C until used. The protein concentration of the liver homogenates was determined as described in Section 3.4.4.

The conjugated diene content of rat livers were determined in duplicate according to the method by Hu *et al.* [61] with slight modifications. Chloroform/methanol (1:2, *v*/*v*) extracts of liver homogenates were prepared, evaporated to dryness under a stream of nitrogen, the lipids dissolved in 1 mL hexane and the absorbance recorded at 233 nm. Lipid peroxidation was expressed as nmole conjugated dienes/mg protein.

Lipid peroxidation was determined according to the method of Buege and Aust [62]. An aliquot (containing 1 mg protein) of liver homogenate was pre-incubated with 0.2 mL 2.5 mM Fe(II)SO_4_ for 1 h at 37 °C. Lipid peroxidation was expressed as μmol malondialdehyde equivalents/mg protein.

### 3.5. Gene Expression of Antioxidant Defense and Oxidative Stress Related Genes

#### 3.5.1. Extraction, Cleanup and Quality Assessment of Ribonucleic Acid (RNA)

Total RNA was isolated from the livers of five randomly selected rats for each of the four treatment groups, using the QIAGEN RNeasy mini kit according to the manufacturer’s specifications. The livers were thawed on ice and 20–25 mg of each sample was lysed in 600 μL of a highly denaturing guanidine isothiocyanate (GITC)-containing lysis buffer (RLT), followed by homogenizing in a QIAGEN Tissuelyser II (Separations, Cape Town, South Africa) for 40 s. RNA was eluted in 50 μL RNase-free water. The Ambion^®^ Turbo DNA-free™ kit (Applied Biosystems, Johannesburg, South Africa) was used to remove contaminating genomic DNA according to the manufacturer’s recommendations. The RNA quantity and purity were determined, using a NanoDrop^TM^ 1000 spectrophotometer (Thermo Fisher Scientific, Wilmington, DE, USA) and RNA integrity was confirmed by determining the RNA 28S:18S ratio, using a Bioanalyzer 2100 (Agilent Technologies, Johannesburg, South Africa).

Following the RNA quality assessment, samples (isolated RNA) were stored at −80 °C prior to gene analyses. Of the five randomly selected samples of each treatment group, three RNA samples were selected for further evaluation with RT^2^-Profiler PCR Array analyses based on RNA integrity parameters.

#### 3.5.2. cDNA Synthesis

First strand complimentary deoxyribonucleic acid (cDNA) synthesis was carried out using the RT^2^ First Strand Kit (SABiosciences, Whitehead Scientific, Cape Town, South Africa) following the manufacturer’s protocol. Contamination of genomic DNA was further eliminated from total RNA (1 μg) with 2 μL of a five times concentrated genomic DNA elimination buffer and nuclease-free water to a final volume of 10 μL and incubated at 42 °C for 5 min. The sample was subsequently mixed with oligo-dT primers, a five times concentrated reverse transcription buffer and reverse transcriptase to a final volume of 20 μL. Following incubation at 42 °C for 15 min the RT reaction was terminated by heating at 95 °C for 5 min and the mixture stored at −20 °C until analyzed.

#### 3.5.3. Quantitative Real Time-PCR Array Analysis

The RT^2^ Profiler Array was used according to the manufacturer’s specifications to evaluate the expression of 84 oxidative stress and antioxidant defense genes (list of genes included available from the manufacturer. A volume of cDNA (102 μL) was combined with 550 μL RT^2^ SYBR Green Mastermix (Qiagen/SABiosciences) that contains HotStart DNA Taq Polymerase (Whitehead Scientific, Cape Town, South Africa). Samples were pipetted into 384-well PCR Array plates (SABiosciences) embedded with primers encoding for 84 genes involved in oxidative stress and antioxidant defense mechanisms (Whitehead Scientific, Cape Town, South Africa). Analysis included a total of two plates, including liver samples from three rats for each treatment group (GRE and control). The array contains 84 primer pairs of oxidative stress and antioxidant defense pathway-focused genes and five primers of reference genes: ribosomal protein large P1 (*Rplp1*), hypoxanthine guanine phosphoribosyl transferase (*Hprt*), ribosomal protein L13A (*Rpl13a*), lactate dehydrogenase A (*Ldha*) and β-actin (*Actb*). Seven wells were used to test non-transcribed genomic DNA contamination and PCR performance. Each sample was tested in triplicate.

Preparation involved dilution of 20 μL cDNA in nuclease-free water to a final volume of 111 μL. A sub-sample (102 μL) was mixed with a two times concentrated RT^2^ qPCR SYBR green I master mixture (550 μL), containing HotStart DNA polymerase, and nuclease-free water (448 μL) to a final volume of 1100 μL. For analysis 10 μL of the cDNA mixture was transferred to a PCR profiler array containing 84 genes, coding oxidative stress and antioxidant defense genes, five reference genes and quality control parameters. Data were generated in real time from the ABI 7900HT real time PCR machine (Applied Biosystems, Johannesburg, South Africa) with a two-step cycling program (1 cycle at 95 °C for 10 min; 40 cycles at 95 °C for 15 s and then at 60 °C for 1 min). Data were analyzed using the Excel-based PCR array data analysis template from the SABiosciences website:

#### 3.5.4. Data Normalization and Analysis

The mean Ct value was determined for each cDNA sample by calculating the difference in Ct between the target gene and reference gene. The comparative Ct (ΔΔCt) method was used to calculate the relative amount of transcripts in the treated and untreated samples (control). The fold change for each treated sample relative to the control sample (obtained from rats receiving no tea treatment) was calculated using the formula of 2^−ΔΔCt^.

### 3.6. Statistical Analysis

Statistical analyses were performed using NCSS 9 Statistical Software (NCSS, LLC, Kaysville, UT, USA). Initial One-way analyses of variance (ANOVA) were performed on all the variables, untransformed and log transformed, to determine whether the within treatment residuals were complying with the assumptions for the analyses. Thereafter, General Linear Model ANOVAs were performed on the untransformed or log transformed data, where applicable as indicated in the first analysis. The Kramer-Tukey multiple comparison tests, at alpha = 0.05, were performed to compare the means. Statistical calculations for gene expression data were conducted based on the ΔCt values using a two-tailed *t*-test. A *p*-value equal to and/or less than 0.05 (*p* ≤ 0.05) was designated as significant.

## 4. Conclusions

The current study indicated that dietary GRE exhibited limited adverse effects in rats when administrated for 28 and 90 days in two separate studies, implying that the dose and duration of exposure to polyphenol-enriched extracts should be carefully monitored. The subtle disruption of liver function is likely to impact on biliary function affecting cholesterol metabolism. The disruption of the GSH redox cycle due to polyphenol/GSH interactions will have a major impact on the cellular redox status as well as the xenobiotic metabolism in terms of the normal physiology and cellular defense against xenobiotic insults. The use of blood biochemical and oxidative parameters during the safety assessment of polyphenol-enriched plant extracts, functional foods or nutraceuticals is, however, questionable due to their lack of sensitivity and specificity. More sensitive approaches such quantitative real time-PCR array analysis of the antioxidant defense and reactive oxygen genes and the level and activity of their respective proteins may be required to provide a more thorough evaluation. In conclusion, the disruption of oxidant/antioxidant homeostasis in a cell by polyphenol-enriched extracts is a double-edged sword when considering their beneficial and/or adverse biological effects.
