# Correction: Van der Merwe, J.D., *et al*. Short-Term and Sub-Chronic Dietary Exposure to Aspalathin-Enriched Green Rooibos (*Aspalathus linearis*) Extract Affects Rat Liver Function and Antioxidant Status. *Molecules* 2015, 20, 22674–22690

**DOI:** 10.3390/molecules21070907

**Published:** 2016-07-12

**Authors:** Johanna Debora Van der Merwe, Dalene de Beer, Elizabeth Joubert, Wentzel C. A. Gelderblom

**Affiliations:** 1 Department of Food Science, Stellenbosch University, Private Bag X1, Matieland (Stellenbosch) 7602, South Africa; debora@factssa.com (J.D.M); JoubertL@arc.agric.za (E.J.); 2 Post-Harvest and Wine Technology Division, Agricultural Research Council (ARC), Infruitec-Nietvoorbij, Private Bag X5026, Stellenbosch 7599, South Africa; DBeerD@arc.agric.za; 3 Institute of Biomedical and Microbial Biotechnology, Cape Peninsula University of Technology, P.O. Box 1906, Bellville 7535, South Africa; 4 Department of Biochemistry, Stellenbosch, Private Bag X1, Matieland (Stellenbosch) 7602, South Africa

The authors wish to make the following corrections to their published paper [1]. Values for the daily intake of GRE extract, total polyphenols and aspalathin in Tables 1 and 2 and the text, including the Abstract, are 10 times too high as they were calculated incorrectly on the basis of 2 g extract/100 g feed mixture instead of 2 g extract/kg feed mixture. Consequently, HED values were also 10 times too high.

These changes do not affect the context of the discussion, except for comparison of TP intake values with that from a 10 week study of rats, drinking unfermented rooibos. The text on p. 22681 should read “dietary intake of 6.2 mg GAE/100 g bw/day for the current study was 2.5 times lower than that of the ten week study when unfermented rooibos was consumed (16.2 mg GAE/100 g bw/day). The TP exposure for 10 weeks increased the GSH level while in the current study, the lower dose over a period of 90 days reduced the GSH level.”

The authors would like to apologize for any inconvenience caused to readers by these changes.
